# Virtual Deformable Image Sensors: Towards to a General Framework for Image Sensors with Flexible Grids and Forms

**DOI:** 10.3390/s18061856

**Published:** 2018-06-06

**Authors:** Wei Wen, Siamak Khatibi

**Affiliations:** Blekinge Institute of Technology, 37179 Karlskrona, Sweden; wei.wen@bth.se

**Keywords:** framework, sensor grid, pixel form, hexagonal, Penrose, deformable sensor, HoG

## Abstract

Our vision system has a combination of different sensor arrangements from hexagonal to elliptical ones. Inspired from this variation in type of arrangements we propose a general framework by which it becomes feasible to create virtual deformable sensor arrangements. In the framework for a certain sensor arrangement a configuration of three optional variables are used which includes the structure of arrangement, the pixel form and the gap factor. We show that the histogram of gradient orientations of a certain sensor arrangement has a specific distribution (called ANCHOR) which is obtained by using at least two generated images of the configuration. The results showed that ANCHORs change their patterns by the change of arrangement structure. In this relation pixel size changes have 10-fold more impact on ANCHORs than gap factor changes. A set of 23 images; randomly chosen from a database of 1805 images, are used in the evaluation where each image generates twenty-five different images based on the sensor configuration. The robustness of ANCHORs properties is verified by computing ANCHORs for totally 575 images with different sensor configurations. We believe by using the framework and ANCHOR it becomes feasible to plan a sensor arrangement in the relation to a specific application and its requirements where the sensor arrangement can be planed even as combination of different ANCHORs.

## 1. Introduction

**Figure 1:**
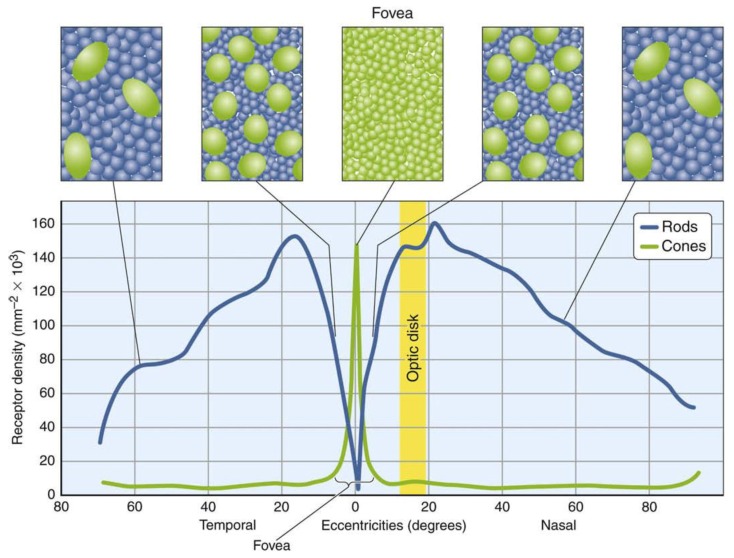
Distribution of photoreceptors in retina of the eye [9].

The most dominant grid structure of the image sensor in a digital camera is the two-dimensional square grid, where each pixel has a square as its basic form. The ease of its implementation in the Cartesian coordinate system is the main reason for its popularity since the invention of the first digital image camera. In recent years, the performance of digital cameras has improved drastically due to increase of resolution of the image sensors achieved by reducing the pixel size. In some special image sensors such as OV5675 from OmniVision [1], the pixel size is as small as 1.12 μm×1.12 μm. However, the smaller pixel size results to lower dynamic range (DR), lower signal-to-noise ratio (SNR) and lower fill factor (FF) [2], indicating that by reducing the pixel size the image quality is reduced. Moreover, the optical diffraction limit; which is a constraint by the aperture of optical elements, makes it impossible to physically reduce the pixel size less than 1.22 λf/D according to Rayleigh criterion, where λ is the wavelength of light, f is the focal length of lens, and D is the aperture diameter. The wavelengths of visible light range are between 390 nm to 780 nm for a typical human eye. The Quanta Image Sensor (QIS) is proposed to overcome the sub-diffraction-limit [3,4,5], where each pixel is partitioned into thousands of single photon sensitive sub-pixels (e.g., 200–1000 nm pitch) referred to as “jots”. The sensor measures light intensity using oversampled binary observations which is sensitive to single photon. Its architecture allows high spatial (>109/sensor) and temporal resolution (>102–103 Hz) of photon strikes on image planes. However, image reconstruction of the sensor remains a challenging issue. Anatomical and physiological studies indicate that our visual quality-related issues, such as high contrast sensitivity, high SNR, and optimal sampling are related directly to the form and arrangement of the sensors in the visual system [6], which have a significant role in optimizing the visual acuity [7]. Within the retina of our eye, the photoreceptor (the rods and cones) mosaic determines the amount of information which is retained or lost by the sampling process, including resolution acuity and detection acuity [8]. The photoreceptor layer specialized for maximum visual acuity is in the center of the retina; i.e., the fovea. Figure 1 shows the distribution of photoreceptors in the areas from the center to the periphery of the retina. The shape of cones in the fovea is similar to a hexagonal structure, densely packed, with virtually no gap between neighboring cones. However, in the periphery, the cones and rods are not packed closely, particularly the cones are far apart from each other, and the form of each photoreceptor is closer to elliptical.

Inspired by the visual system, the hexagonal grid structure has been proposed and implemented on hardware, since many years ago, as an alternative grid structure for image representation instead of the conventional grid, due to its advantages compared to the square grid [10,11,12]. Two examples of such hardware implementations are the super CCD from Fujifilm which features an octagonal-formed pixel in a hexagonal sensor grid [13], and color filters in hexagonal form for the image sensors to improve the quality of the acquired information by the sensor [14]. The cost of transferring the popular square grid and pixel form to the hexagonal ones in camera and display technologies has been one of the issues in today’s unpopularity of the hardware hexagonal technique implementation. The other issue is the difficulty of image processing of hexagonal images where unlike the square grid, the points in a hexagonal grid do not easily lend themselves to be addressed by integer Cartesian coordinates; due to that the points are not aligned in two orthogonal directions. Besides hardware implementation of the hexagonal grid, several attempts at building artificial retinas with electronic hardware are also achieved showing a development from implementations with discrete components [15] over first integrated versions [16] to high-density arrays [17] with resolutions of up to 48,000 pixels [18]. However, when it is about flexibility, once one image sensor is manufactured, it is almost impossible to change the sensor grid and pixel form physically on the hardware. To overcome the hardware limitation and deform the current image sensor closer to the retina, a software-based framework is necessary to achieve an optimal spatial sampling process. Such a framework can offer flexibility in design of pixel form and grid structure of the image sensor.

Numerous software solutions using image processing algorithms were developed to generate a virtual image sensor. Based on the grid pattern, there are two types of the virtual sensor grids: periodic tiling grid (i.e., the square or hexagonal grids) [7] and aperiodic tiling grid (i.e., Penrose or log-spiral grids) [19,20]. Currently, generation of the hexagonal or Penrose sensor grid is generally achieved by having larger pixels with linear and non-linear interpolation of intensity values of the square pixel form in the Cartesian system, such as the nearest neighbor, bilinear, bicubic and the spline based on least-squares [21,22,23]. In different tiling grids, there are various pixel forms which can be fixed and regular, such as square, hexagonal and rhombus, or can be dynamic and irregular, such as Voronoi [24,25]. To have a higher fill factor, all the pixel forms are used for removing the gap between pixels. However, the achievement of the different pixel forms in different grids are still done by interpolation.

In this paper, we propose a general framework towards a virtual deformable image sensor in which grid, pixel and the gap (i.e., the empty space among pixels) can change their form and size. This facilitates application-based configuration of grid, pixel, and gap on the image sensor. In the core framework, we use the idea of modelling the incident photons onto the sensor surface which is elaborated in our previous works [26,27]. Accordingly, each pixel of a captured image by a traditional image sensor (i.e., having square grid and pixel form) is projected onto a grid of L × L square subpixels in which the grid is arranged by the known fill factor or its estimation value as in [28]. Inspired by Monte Carlo simulation, the intensity values of the subpixels are estimated by a statistical resampling process; using a local learning model, a Bayesian inference method, and a maximum likelihood estimator (of Gaussian distribution). Then the subpixels are projected onto the deformed pixel and grid of image sensor; based on the grid and pixel configuration. Certain configurations are studied in our previous works:

(a) The grid and pixel are square and there is no or fixed gap [26,27]. By virtual increase of the fill factor to 100%, the gap between actual pixels in a CCD camera sensor is removed. The results show the dynamic range is widened and tonal level is extended.
(b) The grid and pixel are hexagonal and square respectively and there is no or fixed gap in [29], where the hexagonal grid is generated by a half-pixel shifting, its results show that the generated hexagonal images are superior in detection of curvature edges to the square images.
(c) The grid and pixel are hexagonal and there is no gap [30]. In this work, the impact of the three sensor properties, the grid structure, pixel form and fill factor, is examined by curviness quantification using gradient computation. The results show that the grid structure and pixel form are the first and second most important properties and the hexagonal image is the best image type for distinguishing the contours in the images.
  In this study we pay attention to two new configurations:
(d) The grid and pixel are hexagonal and there is a fixed gap;
(e) The grid and pixel are Penrose and there is no or fixed gap. In this paper, the feature descriptor, histogram of oriented gradient (HoG), is used for examining the impact of the above two configurations to obtain the characteristics of the sensor structure.

This paper is organized as follows: in Section 2 and Section 3, periodic and aperiodic tiling on an image sensor and the methodology are discussed in more detail. In Section 4 the implementation of HoG in relation to the configurations of d and e is elaborated. Section 5 presents the experiment setup. Then the results are shown and analyzed in Section 6. Finally, we summarized and discussed our work in this paper in Section 7.

## 2. Virtual Deformable Image Sensor

**Figure 2:**
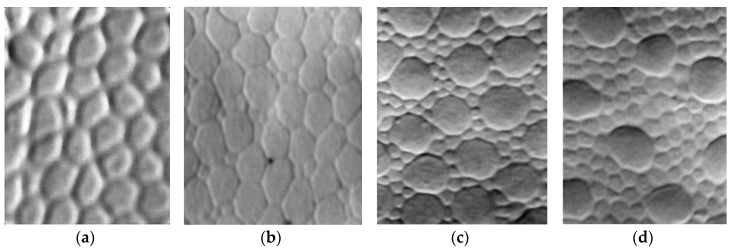
The enhanced and zoomed images of four segments of a human foveal photoreceptor mosaic from the original image printed in [8], From left to right, the segment is chosen from (**a**) the center of fovea, (**b**) the slope of fovea, and (**c**,**d**) the peripheral areas that are 1.35 mm and 5 mm away from the fovea center respectively.

**Figure 3:**
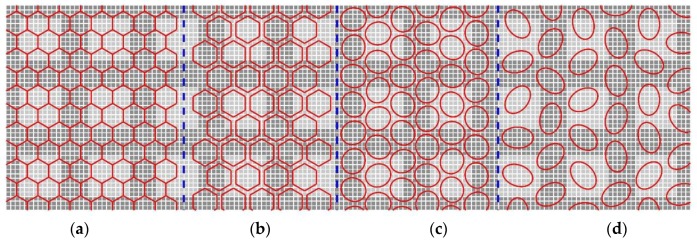
A simulated sensor structure according to the distribution of photoreceptors in retina of the eye. Each area of (**a**–**d**) corresponds to each segment of (**a**–**d**) in Figure 2.

The enhanced and zoomed images of four segments of a human foveal photoreceptor mosaic from the original image printed in [8] are shown in Figure 2. From left to right, the segment is chosen from the center of the fovea, the slope of the fovea, and the peripheral areas that are 1.35 mm and 5 mm away from the fovea center, respectively. In the fovea center, the photoreceptors are only cones and packed densely, the form of each cone is close to hexagonal in shape. When the cones are farther far away from the fovea center, the cone size is getting larger, and their form is changing from hexagonal to circular, and then to elliptical. Figure 3 shows a simulated sensor structure according to the distribution of cones in retina of the eye shown in Figure 2. The area in Figure 3 is separated into four areas a, b, c and d, each of which corresponds to one segment in Figure 2. The red contours represent the active pixels on the image sensor. From the left to right, the gaps between the pixels are getting larger as well as the pixel size from dense to sparse, and the form of pixels is changing from the hexagon to round and to ellipse. Although densely packed sensors without gaps between pixels have higher visual resolution, the pixel size is smaller, which limits the visual detection, i.e., the presence of a spatial contrast (tonal levels) [31]. To simulate a sensor structure close to the cones distribution in the retina, a framework is necessarily for generating image sensors with different configurations based on grid structure, pixel form and gap. In this paper, we mainly focus on the area a and b shown in Figure 3, where the pixel forms and sensor grids are kept the same with no gap and fixed gap.

The design of the pixel arrangement and pixel form on an image sensor is dependent on tiling, which is a way of covering a flat surface with smaller forms or tiles without gaps or overlaps. In an image sensor, each pixel is a tile. When the pixels are repeating themselves in regular and periodic intervals, it will be called periodic tiling. On the contrary, when the pattern of pixels is not repeatable, it will be aperiodic or non-periodic tiling. In conventional image sensor systems, the most familiar tiling is periodic, e.g., by squares pixels, which form the basic building unit of a digital image. Due to the difficulty of physical deformation on image sensors, various virtual sensor grids are generated based on the two types of tiling techniques. Except the general square pixel tiling sensor, hexagonal tiling and Penrose tiling are two most popular representatives for periodic and aperiodic tiling, respectively.

### 2.1. Hexagonal Tiling

**Figure 4:**
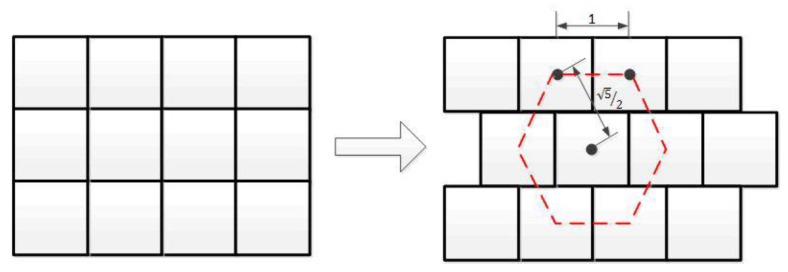
The procedure from square pixels to hexagonal pixels by half-pixel shifting method.

**Figure 5:**
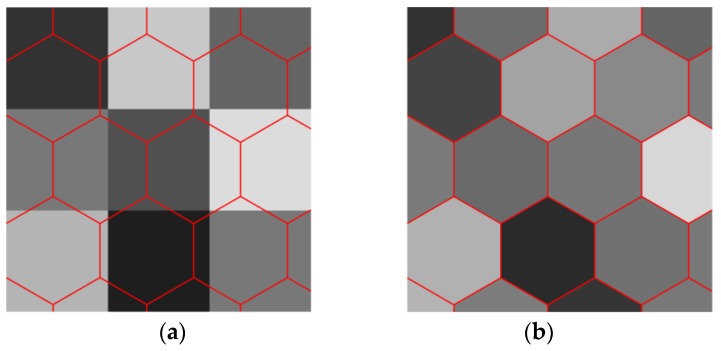
Illustration of the square to hexagonal lattice conversion by the hyperpel method (**a**) the sub-pixels in each surrounded area by red boundary are clustered together for the corresponding hexagonal pixel and (**b**) the value of each hexagonal pixel is the average intensity of the sub-pixels within each cluster [35].

In hexagonal tiling, the hexagonal pixels are arranged in a hexagonal grid. A hexagonal image is generally generated from an original square image. The half pixel shifting method is a popular method in generation of such hexagonal images, which is derived from delaying the sampling by a half a pixel on the horizontal direction as it is shown in Figure 4 [32]. In Figure 4, the left and right patterns are showing the conventional square lattice and the new pseudo-hexagonal sampling structure, whose pixel form is still square; see the six connected pixels by the dashed lines. In such sampling structure, the distance between the two sampling points, pixels, are not the same; they are one or $\sqrt{5}/2$. Another well-known way of hexagonal image generation upsamples firstly the original square lattice data to a much denser square sub-pixels by interpolation [33], and then cluster a group of sub-pixels of the intermediate data together into an equivalent hexagonal pixel. By this way a pseudo hexagonal pixel, known as a hyperpel, is generated from a cluster of square pixels [34], which is widely used for displaying a hexagonal image on normal monitor. In Figure 5b, each area surrounding with red boundary is a cluster of square subpixels represented by a hyperpel. In this method, the distance between each two adjacent hexagonal pixels is almost the same, and the form of each pixel is close to a hexagon. In both methods, the new pixel intensity in the hexagonal grid has the average intensity value of a group of square pixels, as it is done in Figure 4 and Figure 5.

### 2.2. Penrose Pixel Arrangment

**Figure 6:**
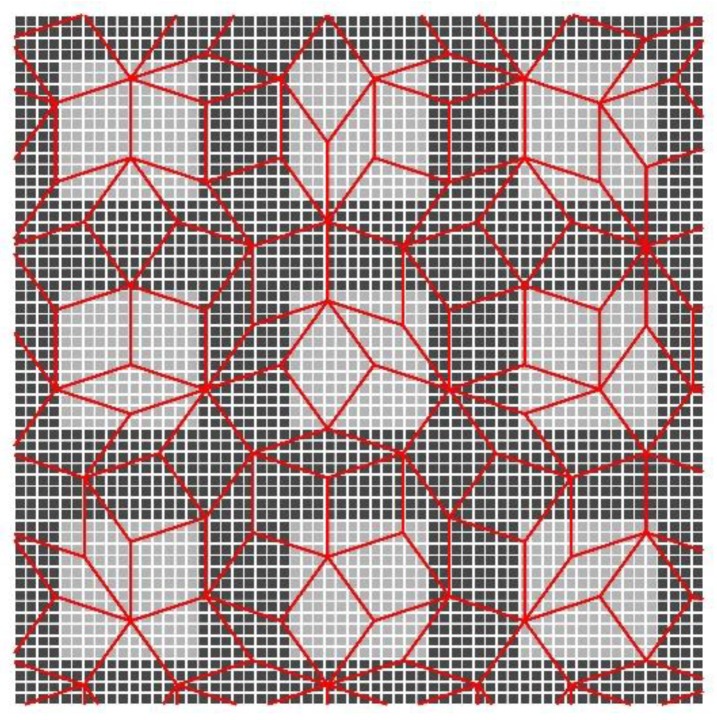
An example of the rhombus Penrose tiling.

Although the hexagonal structure is very close to the true structure of human vision system in the fovea, however it is still far away from a perfect and real model of it. The truth is that photoreceptors in the human fovea are arranged non-periodically, due to that each of photoreceptors as a tile is not repeatable in the region of human retina. One way to construct the aperiodic pixel grid is to use Penrose tiling, which was presented by Penrose in 1973 [36], and has been used for building the models of the sensor pixel layout [24]. Figure 6 shows the rhombus Penrose tiling, which consists of two types of rhombuses, each placed at five different orientations by specific rules [36]. The two types of Penrose rhomb tiles can be divided into two groups of thin and thick rhombuses. The thin rhomb has four corners with angles of 36, 144, 36, and 144 degrees, and the thick rhomb has angles of 72, 108, 72, and 108 degrees. The ratio of the number of thick to thin rhombi is the Golden Number $\frac{1+\sqrt{5}}{2}$, which is also the ratio of their area. Unlike the periodic tiling, Penrose tiling has no translational symmetry; it never repeats itself exactly. This means that it is theoretically possible to integrate and sample the infinite plane indefinitely without repeating the same pixel structure. In practice, this allows to virtually sample a significantly larger number of different images than is possible with a regular, square grid. In the method presented in [24], the Penrose tiling is achieved by the process of upsampling and resampling. The upsampling is done by placing the regular pixel intensities over a new grid of square subpixel and implementing the nearest neighbor interpolation. Then the new grid of subpixels is projected onto the grid of Penrose pixels. Each of Penrose pixel, the thin or thick rhombus, is composed by a cluster of subpixels. The Penrose pixel intensity is the average intensity value of the subpixel intensities that belong to its cluster.

Although the virtual sensor can be generated with hexagonal or Penrose structures; i.e., by interpolation means as it was discussed above, however the problems related to the upsampling process are remained in these arrangements. We believe by implementation of reconstruction instead of interpolation process it is possible to solve this challenging problem. In a reconstruction process new data (i.e., for the non-available subpixels intensities) is added to the current one (i.e., the available subpixels intensities) using a resampling model; e.g., a model based on incidental photons onto sensor surface. An interpolation process is using the current data to predict non-available subpixels intensities; e.g., by using a local mean filter. Thus, it is reasonable to use the reconstruction process in a framework of a deformable sensor grid which it is elaborated in Section 3.

## 3. Image Generation on Deformable Grid

In this section, the process of generating an image using the virtual deformable sensor under the framework is explained. According to the model of the incident photons onto the sensor surface presented in [26], the reconstruction process is divided into three steps of: (a) projecting each original pixel intensity onto a new grid of subpixels based on the gap size and form in the configuration; (b) estimating the values of subpixels based on a local learning model; and (c) estimating the new pixel intensity by decision-making based on the grid structure and pixel form in the configuration. The three steps are elaborated below:

(a) A grid of virtual image sensor pixels is constructed. Each original pixel, having square pixel form and arranged in square grid is projected onto a grid of L × L square subpixels. According to the configuration size of the gap G between the pixels, the size of the active pixel area is defined as S×S, where S=L−G. The intensity value of every pixel in the image sensor array is assigned to the virtual active pixel area in the new grid. The intensities of subpixels in the gap areas are assigned to be zero. An example of such sensor rearrangement on sub-pixel level is presented in Figure 6, where there is a 3 by 3 pixels’ grid, and the light and dark grey areas represent the active pixel areas and the gap areas. Assuming L=30 and S=18, and thereby the gap size becomes G=12 according to the above equation.
(b) The second step is to estimate the values of subpixels in both pixel areas and gap areas. Considering the statistical fluctuation of incident photons and their conversion to electrons on the sensor is a random Gaussian process, from a certain neighborhood area of each pixel, a local Gaussian model is generated by maximum likelihood method. Then a local noise source is generated within each local model, and introduced to its certain neighborhood. Inspired by Monte Carlo simulation, all subpixels in each certain neighborhood are estimated in an iteration process using the known pixel values (for subpixels in the active pixel area) or by linear polynomial reconstruction (for subpixels in gap area). In each iteration step the number of subpixels in the pixel area is varied from zero to total number of subpixels in pixel area. After the iteration process, a vector of intensity values for each subpixel is generated and the final subpixel value is predicted using Bayesian inference method and maximum likelihood of Gaussian distribution.
(c) In the third step, the subpixels are projected onto the new deformable sensor grid with different sensor grid, pixel form and gap size in respective configuration proposed in Section 2. In this paper, three sets of configurations are considered: (1) square grid and pixel form with or without gap; (2) pseudo-hexagonal grid by half-square-pixel shift and square pixel form with or without gap; (3) hexagonal grid and pixel form with or without gap; and (4) Penrose grid and rhombus pixel form with or without gap, where each of the configurations deformability is demonstrated. For the image generation in the different grids, the subpixels are projected back onto the new sensor grid. The intensity value of each pixel in different sets of configurations is the intensity value which has the strongest contribution in the histogram of its belonging subpixels.

## 4. Implementing Histogram of Gradient in Different Configurations

**Figure 7:**
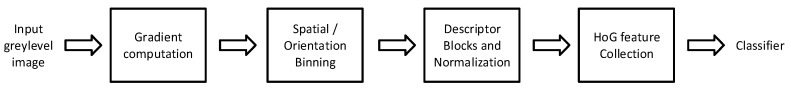
An overview of the feature extraction and object detection chain.

In [30], the gradient is proved to be an effective parameter for examining the impact of different sensor grids and pixel forms on curviness. In this paper, the histogram of gradient (HoG) is used for evaluating the characteristic of the sensors having different configurations. The general process of HoG on square images; i.e., from extracting features to object detection, is presented in Figure 7, which is divided into four steps. The first step is gradient computation. In [37], it is proved that the performance of feature detection is sensitive to the way in which gradients are computed, but the simplest scheme turns out to be the best. The most common method is to apply the one dimensional centered, point discrete derivative mask in both horizontal and vertical directions with the filter kernels of ${\left[-1\;0\;1\right]}^{T}$ and [−1 0 1] . The second step in HoG process is spatial/orientation binning. In this step, the image is divided into a group of cells, each of which is a local spatial region in the image. For pixels within each cell a histogram of gradient directions is compiled where each pixel casts a weighted vote (typically as the gradient magnitude itself), for an orientation-based histogram based on the computed gradient values and then the votes are accumulated into orientation bins over the cell. Cells can be either rectangular or radial in shape. The orientation bins are evenly spread over 0 to 180 degrees (unsigned gradient) or 0 to 360 degrees (signed gradient). In the third step in HoG process due to the local variations in illumination and foreground-background contrast, the gradient strengths are normalized to reduce the variation. The effective local contrast normalization turns out to be essential for good performance [37]. The current normalization methods mostly are based on grouping cells into larger spatial blocks and contrast normalizing each block separately. The main geometries of the blocks are rectangular R-HoG blocks [37], circular C-HoG blocks [38] and hexagonal H-HoG blocks [39]. According to the result in [39], the hexagonal structure block is more effective and efficient than conventional structure block. The fourth and final step in HoG process is generation of feature descriptor which is a concatenated vector of all components of the normalized cell histograms from all the blocks.

Due to the fact that the sensor arrangements of hexagonal and Penrose images are hexagonal and aperiodic, respectively, the gradient computations in these arrangements are different from the square arrangement where the HoG is generally implemented. The hexagonal grid is periodic tiling, and each of its pixel has six adjacent neighbor pixels within the same distance, which is unlike the pixel in the square grid which has four neighbors; i.e., the gradient computation in hexagonal grid is done in three directions with the same kernel of [−1 0 1].

The gradient computation in Penrose grid is more complex due to the fact the pixels are arranged by aperiodic tiling, which the kernel of [−1 0 1] cannot be implemented directly. In a Penrose grid, each pixel has four adjacent neighbor pixels. Each pixel has the form of a thin or thick rhombus with two pairs of 36 and 144 or 72 and 108 degrees, respectively. The slope of the line which connects two corners of such rhombi with a smaller pair of angles is used as pixel direction. The directions of each pixel and its adjacent pixel are used to obtain five virtual vectors with the respective direction from the same origin where the magnitude of vectors are the intensity values of the respective pixel. The gradient between an actual pixel and each of its neighboring ones is computed by vector subtraction of the respective vectors which allows us to find the orientation and amplitude of the gradient.

## 5. Experimental Setup

**Figure 8:**
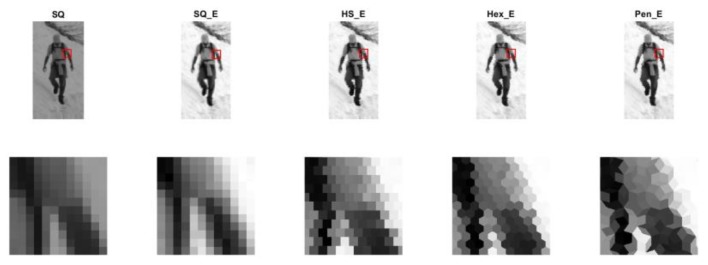
One of original images and its set of generated images.

The dataset used in the experiment is ‘INRIA’ proposed in [37], which contains 1805 cropped images of humans taken from different sets of personal photos. This dataset was produced at beginning for the challenging task of pedestrian detection. The people are usually standing, but appear in any orientation and against a wide variety of background scenery, including crowds. Each of the cropped human images has a resolution of 64 × 128. For the experiment 23 images were randomly selected from this dataset. Each selected image is used to generate four sets of images by implementing the process described in Section 3. The four sets differ due to the type of grid structure which can be square, half pixel shift, hexagon, or Penrose type. We named the generated images in each set based on the type of grid structure, i.e., four sets of square enriched (SQ_E), half pixel shift enriched (HS_E), hexagonal enriched (Hex_E), and Penrose enriched (Pen_E) images. The “enriched (E)” in the name of images refers to tonal enrichment property of images which is obtained by the processing, in comparison to non-processed original type of images. The images of each four sets differ due to the configuration parameters of pixel form and gap size. Figure 8 shows one of the original images (SQ) from the database and one image of each four sets of images. From left to right, the types of images are SQ, SQ_E, HS_E, Hex_E, and Pen_E. The whole images are shown in the first row and the areas marked with red square in each image in the first row are zoomed out and shown in the second row. For visualization purposes each pixel in the hexagonal and Penrose grids is composed by a group of square pixels. The generated images show better dynamic range and higher contrast in comparison to the original images. All the processing is programmed and done by Matlab2017b on a stationary computer with an Intel i7-6850k CPU (Intel Corporation, Santa Clara, CA, USA) and a 32 GB RAM memory to keep the process stable and fast.

## 6. Results and Discussion

**Figure 9:**
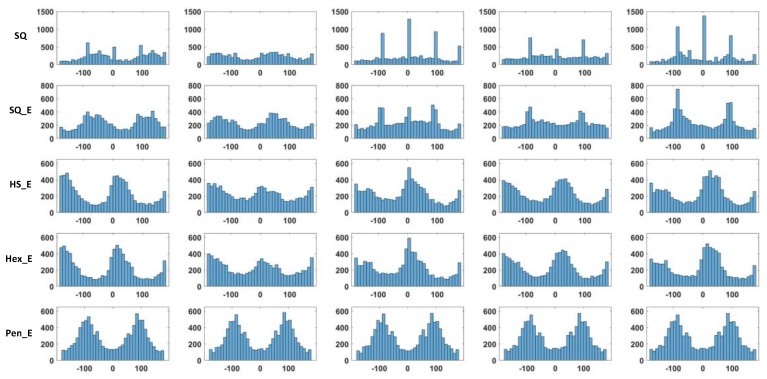
The angular characteristic of sensor grid structure. The five columns of figures are the histograms of the gradient orientation from five images in the database. From the first to the fourth row, the five image types are SQ, SQ_E, HS_E, Hex_E and Pen_E.

For each image of the four sets of images HoG is computed as explained in Section 4. As the gradient indicates the directional change of intensities in an image, the gradient orientation is used to show the difference among different image types. Figure 9 shows row-wise histogram of the gradient orientation of five images of the database and column-wise image types of SQ, SQ_E, HS_E, Hex_E and Pen_E. In the figure considering the results of SQ and SQ_E image types, the peaks of histogram of the gradient orientation are close to 0, 90 and −90 degrees, indicating that square sensor structure is more sensitive to the vertical and horizontal changes. When the sensor structure is hexagonal; having HS_E and Hex_E image types, the result of the two types are very close to each other and for both types there are more sensitive angles in comparison to the square arrangement; the peaks are at 60, 120 and 180 degrees. When Penrose structure images are considered, due to their having two types of rhombus in the pixel form where the angle between each pixel is either around 72 or 144 degrees, the peaks are also close to 72 or 144 degrees. The results related to the five images of Figure 8 are verified for all images of the experimental dataset. Accordingly, the histogram of gradient orientation shows a specific distribution related to each sensor structure with certain gap size. We call this specific distribution as the ANgular CHaracteristic of a sensOR structure (ANCHOR). When the number of bins for orientation of the gradient is 36, the envelope of a histogram becomes smooth as a curve.

**Figure 10:**
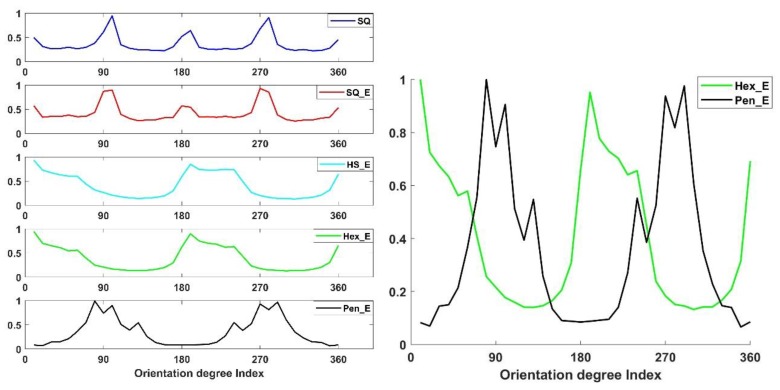
The histograms of the gradient orientation with 36 bins are shown in the (**left**). From top to bottom, there are results related to SQ, SQ_E, HS_E, Hex_E and Pen_E respectively. The ANCHORs show the average values of 23 histograms of the gradient orientation. The (**right**) shows the comparison between ANCHORs of Pen_E (black) and Hex_E (green). These ANCHORs together, representing combination of two types of sensor arrangements, compensate each other’s week sensitivity areas and become more sensitive to detect directional changes of intensities.

In Figure 10, from top to bottom, the five ANCHOR curves show the normalized average values of 23 histograms of the gradient orientation. The result is consistent to what we conclude from Figure 9. The right Figure 10 shows the comparison between ANCHORs of Pen_E (black) and Hex_E (green). These ANCHORs together, representing combination of two types of sensor arrangements, compensate each other’s week sensitivity areas and become more sensitive to detect directional changes of intensities. Part of the mosaic of photoreceptors in the human retina has such a combination of the two types of arrangement.

**Figure 11:**
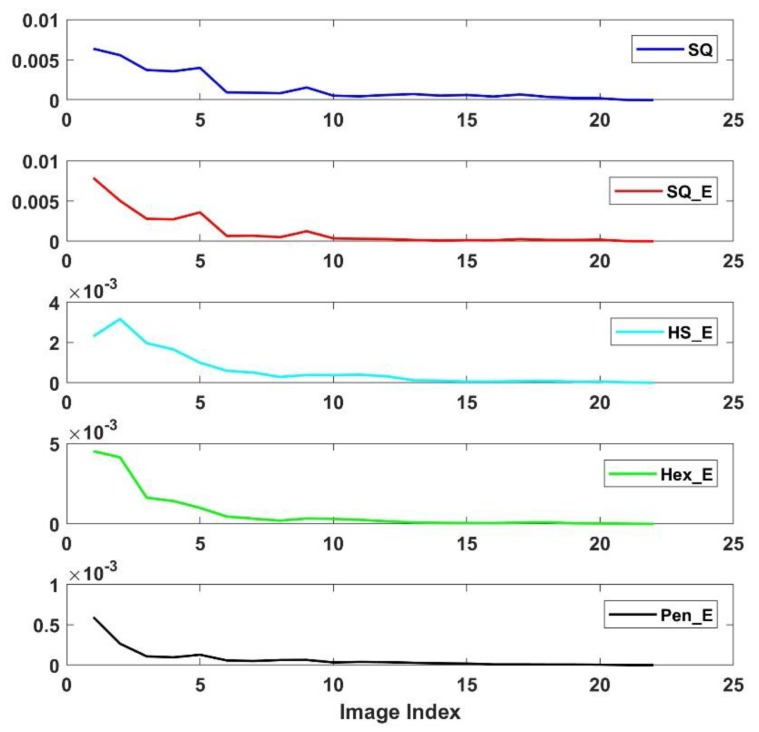
MSE results between each two candidates of certain ANCHOR. Each of the candidates are computed as average values of orientation of the gradient for certain number (*n*) of images.

**Figure 12:**
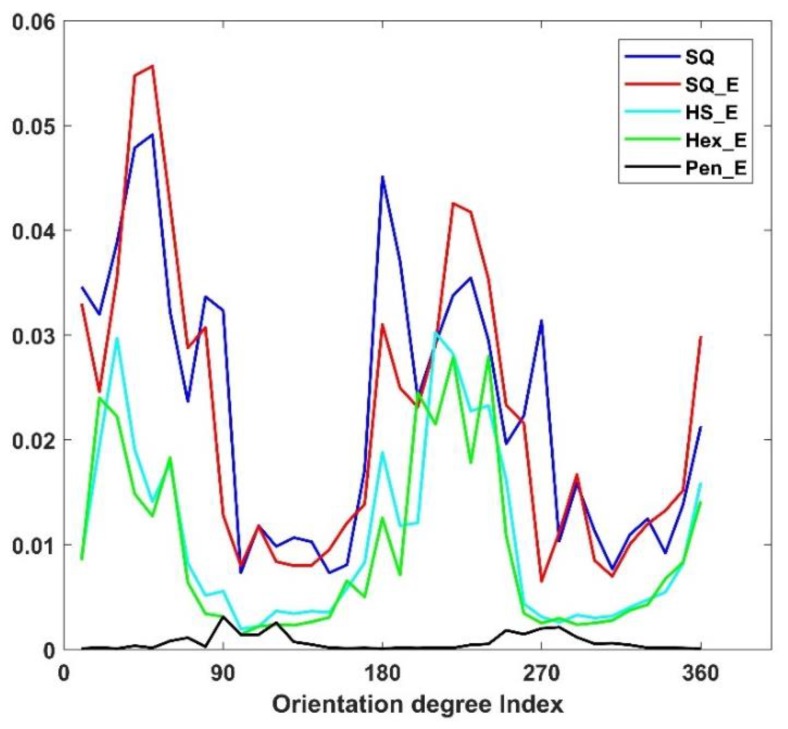
The variance of ANCHORs is demonstrated. The histograms of the gradient orientation with 36 bins are used and from top to bottom, there are results related to SQ, SQ_E, HS_E, Hex_E and Pen_E respectively. The ANCHOR of Pen_E (black) has the lowest variance and the SQ (deep blue) have the highest, indicate the Penrose arrangement has more robust ANCHOR in comparison to the others.

The characteristic robustness of ANCHORs is examined by computing averaging of n number of histograms of orientation, where n is varied from two to twenty-three; i.e., the computations result to obtain n number of candidates for each ANCHOR. Then the Mean Square Error (MSE) between each two candidates of certain ANCHOR is computed by:

$$MSE=\frac{1}{n}\overset{n}{\underset{i=2}{\sum }}{\left(Y_{i}-\bar{Y}\right)}^{2}$$

where *i* and *n* represent the number of orientation degree index and the number of the averaged histograms respectively. $Y_{i}$ and $\bar{Y}$ represent the average value from i histograms and 23 histograms. The results for different sensor structure are shown in Figure 11, where the five color lines from top to bottom represent five image types, SQ, SQ_E, HS_E, Hex_E and Pen_E. The figure shows that each of MSE result decreases close to zero after ten combinations; indicating no further changes on the respective ANCHOR has occurred. Thus, ten images are enough to obtain a robust ANCHOR for each type of arrangement. As the results in the figure show, the MSE values reduce to small values after having just two images which indicate that the robustness of ANCHORs is strong and almost independent of the number of images. Among ANCHORs, the one of Pen_E has the lowest MSE values, indicating the strongest robustness of the corresponding ANCHOR. The variance of ANCHORS computed from 23 images is shown in Figure 12. It shows that the variance of the arrangement type of Pen_E has the lowest variance values in different orientation angles between 0 to 360 degrees. However, and the ones of the arrangement types of SQ and SQ_E have the highest variance values. The results are consistent with the result in Figure 10, that the Penrose sensor structure has the most robust ANCHOR.

The gap factor is defined in relation to the gap size and pixel size by:

$$gap\;factor=\frac{gap\;size}{pixel\;size}\times 100\%$$

**Figure 13:**
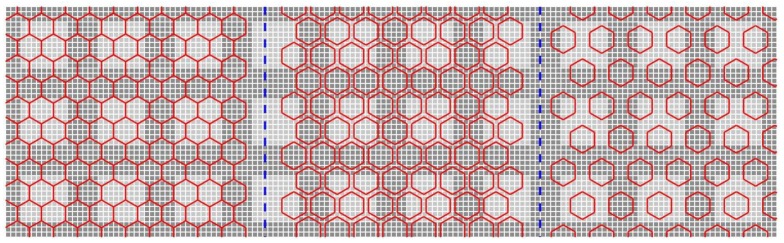
One example of the hexagonal sensor having the gap factor of 0%, 20% and 60% from left to right, and the pixel size is kept the same.

**Figure 14:**
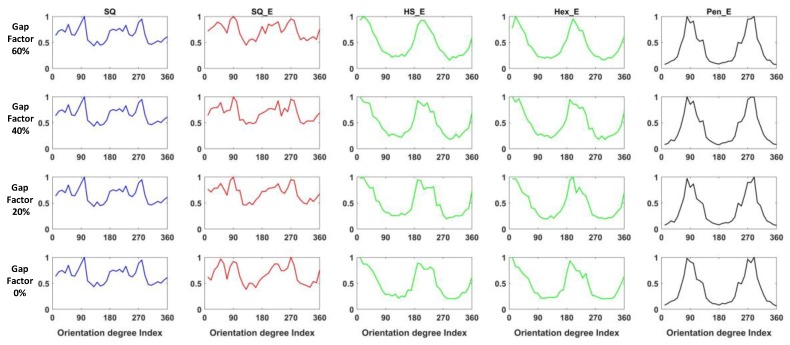
The results of ANCHORs from different types of images with different configurations of gap factor.

Assuming the pixel size is 100 and the gap size between the active areas in two pixels is 20, the gap factor becomes 20%. In the following experiment, the gap size is set at 0, 20, 40 and 60, which is corresponding to gap factor of 0%, 20%, 40% and 60%. Figure 13 shows one example of the hexagonal sensor having the gap factor of 0%, 20% and 60% from left to right where the pixel size is kept the same. Figure 14 shows the results of gradient orientation; i.e., the ANCHORs, of four types of images, SQ_E, HS_E, Hex_E and Pen_E with different configurations of gap factor. In the figure due to that the effect of the gap factors on the SQ image, i.e., the original image, cannot be implemented its ANCHOR is the same for different gap factors. Each column represents one type of images, and the rows represent the effect of different gap factors on the respective ANCHOR. The effect of ANCHOR changes in relation to gap factor is investigated by MSE computation between a reference ANCHOR (having the same pixel size as the original image and gap factor of 0%) and an ANCHOR with the same pixel size but different gap factor in relation to the reference one. The mean of MSEs from 23 images for different types of arrangement are shown in Table 1. In the table the values of average MSEs increase with increase of the gap factor, however the rate of increase is lowest in the SQ_E arrangement type and highest in HS_E and Hex_E ones.

**Figure 15:**
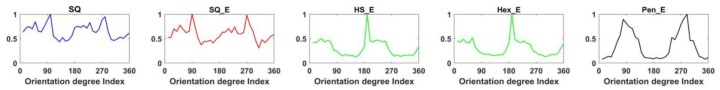
The ANCHORs of different types of images when the pixel size is increased by 20% and gap factor is 0%.

Figure 15 shows the ANCHORs of five types of images (as in Figure 15) when the pixel size is increased by 20% and having gap factor of 0%. All the results show that the images of the same sensor structure follow the same specific pattern. Table 2 shows the MSEs between the reference ANCHOR and ANCHORs of different types of arrangements with increased pixel size of 20% (in comparison to as original image) and gap factor of 0%. The table shows that pixel size increase results in 10-fold impact on ANCHOR of SQ arrangement in comparison to the other types. The Table 1 and Table 2 show that the impact of pixel size changes is greater that gap factor changes on ANCHORs of all involved arrangements.

The HoG is computed for the four set of images in the experiment as explained in Section 4, where each HoG result is represented as a vector. In our experiment, the block size is set to 4, 8 and 16. The correlation between the results of HoG for SQ images and the other four types of images (having the same block size of either 4, 8, or 16) are computed for the similarity comparison which are shown in Table 3. The HoG in SQ_E image has the highest correlation to SQ image because they have the same sensor structure and pixel form. When the block size is increased from 4 to 16, the correlation values increase as well. In Table 3, the three block sizes are marked with three colors of blue, green and red for block size of 4, 8 and 16. In each row, there are four correlation values in relation to each block size, for which the lowest ones are marked blue, green, and red. For example, the lowest value in relation to block size 16 is marked red.

As Table 3 shows, the lowest correlation values related to block sizes are related to the Pen_E and Hex_E arrangements. Such results indicate that one should expect most different results of HoG having Pen_E in first place and then Hex_E arrangements. The differences among the four types of arrangements are measured by using their correlation to the same reference SQ image type. The cause of such differences between each two arrangements is related to the differences between their ANCHORs.

## 7. Conclusions

In this paper, we have presented a framework by which it becomes feasible to create virtual deformable sensor arrangements. In the framework the structure of the arrangement, the pixel form and the gap factor (related to the distance between pixels) are the three optional variables in creation of a sensor arrangement. We showed that the histogram of gradient orientations is a useful tool in measuring an arrangement structure. The envelope of such a histogram is defined as an ordination distribution function. In our experiments we observed that for each image type; having different arrangement structure, the distribution function is unique. Additionally, we showed that a change of pixel size or gap size; i.e., a different sensor configuration, generates a specific distribution related to the changes. We called these specific orientation distributions ANCHORs. We showed that by using at least two generated images of a certain configuration it is possible to obtain the ANCHOR of the sensor. The results showed pixel size changes have more impact on ANCHORs than gap factor changes. By using 575 different configuration images we verified the robustness of ANCHORs and feasibility of the framework, which encourages us to think about the possibility of tailored sensor arrangements in relation to a specific application and its requirements; our results inspire this idea as well. On the right of Figure 10 the ANCHORs of two different sensor arrangements are shown which can be combined to a new sensor arrangement in case of an application requirement for having both the properties of hexagon and Penrose. We believe this idea may result in being able to have a sensor arrangement in the future very alike that of the biological vision sensory system.

## Figures and Tables

**Table 1:** The average MSE of orientation of four image types with different configurations of gap factor referred to the 0% gap factor.

	Referred to 0% Gap Factor			
Gap Factor	SQ_E	HS_E	Hex_E	Pen_E
60%	0.0052	0.0059	0.0078	0.0004
40%	0.0049	0.0029	0.0053	0.00035
20%	0.0050	0.0028	0.0034	0.0003

**Table 2:** The average MSE of orientation of four image types with pixel size increase of 20% and 0% gap factor.

	Referred to 0% Gap Factor			
Gap factor	SQ_E	HS_E	Hex_E	Pen_E
0%	0.0039	0.0308	0.0271	0.0036

**Table 3:** The correlation between the results of HoG for SQ images and the other four types of images in respect to block size.

	Correlation to SQ											
	SQ_E			HS_E			Hex_E			Pen_E		
No.	Size 4	Size 8	Size 16	Size 4	Size 8	Size 16	Size 4	Size 8	Size 16	Size 4	Size 8	Size 16
1	0.593	0.799	0.9	0.123	0.097	0.071	0.096	**0.072**	**0.04**	**0.064**	**0.072**	0.163
2	0.679	0.818	0.907	0.258	0.289	0.341	0.27	0.309	0.369	**0.064**	**0.074**	**0.106**
3	0.627	0.819	0.911	0.155	0.164	0.239	0.149	0.18	0.267	**0.001**	**0.035**	**0.081**
4	0.672	0.809	0.886	0.066	0.131	0.167	0.053	0.12	**0.132**	**0.081**	**0.114**	0.173
5	0.648	0.817	0.929	0.101	0.156	0.097	0.108	0.145	0.107	**0.029**	**0.062**	**0.072**
6	0.676	0.862	0.947	0.074	0.124	0.15	0.066	**0.128**	**0.15**	**0.042**	0.14	0.232
7	0.623	0.822	0.903	0.022	0.111	0.229	0.056	0.126	0.204	**0.012**	**0.015**	**0.067**
8	0.634	0.824	0.912	0.082	0.138	0.107	0.087	0.133	**0.113**	**0.05**	**0.089**	0.121
9	0.65	0.834	0.918	0.01	0.019	0.075	**0.018**	**0.007**	**0.084**	**0.018**	0.076	0.171
10	0.652	0.858	0.951	0.008	0.006	0.14	**0.004**	**0.002**	**0.158**	0.164	**0.243**	0.368
11	0.633	0.802	0.907	0.026	0.042	0.045	0.032	0.059	**0.058**	**0.012**	**0.054**	0.126
12	0.604	0.828	0.912	0.039	0.086	0.078	0.053	0.096	**0.045**	**0.026**	**0.027**	0.105
13	0.634	0.815	0.893	0.146	0.216	0.143	0.145	0.217	0.171	**0.037**	**0.08**	**0.047**
14	0.613	0.789	0.88	0.122	0.19	0.154	0.129	0.189	0.165	**0.006**	**0.007**	**0.01**
15	0.629	0.821	0.877	0.053	0.051	0.001	0.065	**0.038**	**0.006**	**0.013**	**0.038**	0.111
16	0.644	0.81	0.896	0.098	0.111	0.002	0.094	0.093	**0.012**	**0.039**	**0.063**	0.179
17	0.587	0.79	0.889	0.118	0.171	0.072	0.141	0.176	0.122	**0.01**	**0.045**	**0.017**
18	0.612	0.766	0.882	0.063	0.083	0.108	**0.036**	**0.064**	**0.06**	0.096	0.166	0.256
19	0.647	0.809	0.902	0.063	0.133	0.112	0.07	0.124	0.117	**0.036**	**0.026**	**0.027**
20	0.602	0.796	0.885	0.186	0.26	0.275	0.187	0.268	0.284	**0.009**	**0.013**	**0.001**
21	0.636	0.789	0.912	0.027	0.037	0.002	0.029	**0.037**	**0.039**	**0.09**	0.175	0.234
22	0.596	0.795	0.885	0.024	0.055	0.079	0.034	0.045	**0.042**	**0.008**	**0.018**	0.066
23	0.642	0.819	0.894	0.074	0.113	0.17	0.072	0.115	0.132	**0.058**	**0.038**	**0.015**
